# CHANGES IN OLDER ADULTS’ LONELINESS OVER TWO YEARS OF COVID-19: A MIXED-METHODS ANALYSIS

**DOI:** 10.1093/geroni/igac059.2991

**Published:** 2022-12-20

**Authors:** Emily Kinkade, Heather Fuller, Bryce Van Vleet, Andrea Huseth-Zosel

**Affiliations:** North Dakota State University, Fargo, North Dakota, United States; North Dakota State University, Fargo, North Dakota, United States; North Dakota State University, Fargo, North Dakota, United States; North Dakota State University, Fargo, North Dakota, United States

## Abstract

Throughout the COVID-19 pandemic, social distancing and stay-at-home behaviors have been recommended to protect older adults from severe illness. However, despite disease reduction benefits, self-isolation may place older adults at an increased risk for loneliness, another serious public health concern. This longitudinal, mixed-methods study investigates whether older adults’ perceptions of loneliness changed across two years of the pandemic and how older adults described their experiences with loneliness at different points of the pandemic. Between April 2020 and June 2022, five waves of interviews were conducted with 76 Midwestern older adults between the ages of 70 and 97. At each interview, participants completed the UCLA Three-Item Loneliness Scale and answered several open-ended questions about their current daily life, social connections, and experiences and perceptions during the pandemic. A repeated-measures ANOVA indicated that participants’ loneliness scores significantly decreased between the first and final interview, although there was a nonsignificant increase in loneliness at the third interview. Thematic coding of the interview transcripts identified four emergent themes related to loneliness across two years of the pandemic: 1) differentiation of isolation from loneliness, 2) acceptance of circumstances, 3) adaptation to prevent loneliness, and 4) barriers to adaptation. Findings suggest older adults had nuanced and shifting experiences of loneliness throughout the pandemic, in which they generally showed increasing acceptance and strong adaptation to feelings of loneliness. However, adaptation was also challenged as the pandemic endured. Our discussion will highlight protective and risk factors for older adults’ loneliness and directions for future study and practice.

